# Impacts of enrichment programs on cognitive and affective skills of gifted students: A meta-analysis

**DOI:** 10.1371/journal.pone.0333714

**Published:** 2025-10-09

**Authors:** M. Davut Gül, Zekai Ayık

**Affiliations:** 1 Department of Special Education, Tokat Gaziosmanpasa University, Turkiye; 2 Department of Special Education, Harran Unversity, Turkiye; Institute of Medical Biochemistry Leopoldo de Meis (IBqM) - Federal University of Rio de Janeiro (UFRJ), BRAZIL

## Abstract

This meta-analysis aims to study research on enrichment programs for gifted students and synthesize the effects on cognitive and affective skills reported by studies conducted between 2014–2024 years. During the screening process, the focus was on quantitative studies published between 2014 and 2024 that explored the influence of enrichment programs (ER) on cognitive and affective abilities of gifted students. Searches were carried out in Web of Science and Scopus databases for publications in English. The researchers examined publication bias by analysing data using various methods, including the funnel plot, Begg’s test, Egger’s test, and trim-and-fill test. Additionally, the researchers utilized Classic fail-safe N and Orwin’s fail-safe N values for further analysis. Hedges’s *g* was used to report the magnitude or strength of the standardized mean differences between groups in a study as effect size, since it provides effect size for smaller sample sizes. A total of 16 publications included in this meta-analysis, and results demonstrated that enrichment programs had a large effect on cognitive skills (*g = *1.14, 95% CI [.83, 1.45], under random-effect model), a medium effect on affective personal skills (*g* = .51, 95% CI [.30,.73], under random-effect model), and small impact on affective social skills (*g* = .44, 95% CI [.23,.66], under random-effect model). This review underscores nuanced findings focusing on sub-domains of achievement, higher-order thinking, and personal and social-affective skills. The overall results implied that enrichment programs play a significant role in fulfilling both the cognitive and non-cognitive needs of gifted students.

## Introduction

Gifted students demonstrate advanced capabilities in thinking, learning [1–3] and socio-emotional development [4,5]. Although gifted children possess advanced abilities in certain domains, it has been noted that they often experience significant difficulties in coping with challenges in both academic and everyday contexts [6]. Furthermore, they have been reported to face challenges in behavioral and socio-emotional domains as well [7]. Due to their asynchronous development, these children may require educational provisions specifically tailored to their unique needs—needs that are frequently unmet in traditional school environments, yet are essential for fostering their full potential [8,9]. Within the field of gifted education, enrichment programs—designed to offer a deeper and broader exploration of content beyond the standard curriculum—have emerged as key models for addressing these needs [10–14]. In response, a wide range of enrichment models has been developed and implemented globally to support the academic, social, creative, and cognitive development of gifted learners [12,15–19]. These programs are designed to meet the varied needs of gifted students by promoting higher-level thinking and in-depth exploration across multiple domains, including science, technology, engineering, arts, mathematics (STEAM), and leadership. Through a combination of differentiated curricula, extracurricular activities, and specialized instructional strategies, enrichment programs aim to cultivate advanced cognitive skills, creativity, social competence, and personal development [20].

Despite their widespread implementation, there remains a pressing need for enrichment programs that are supported by empirical evidence demonstrating their effectiveness and practical value for gifted education stakeholders, including educators and policymakers [8,12,21]. Research has shown that such programs can significantly enhance students’ academic performance and personal development, often yielding large effect sizes in well-structured and intensive learning environments [8]. While improvements in target skills such as learning strategies or self-concept are typically regarded as indicators of program success, declines in these areas may signal deficiencies in program design or delivery [20]. Meta-analytic research plays a crucial role in assessing the efficacy of enrichment programs by aggregating findings across multiple studies. This method allows for a more comprehensive evaluation of how these interventions influence key developmental areas such as academic achievement, cognitive growth, creativity, and socio-emotional functioning. Moreover, meta-analyses contribute to the identification of best practices and provide evidence-based guidance for shaping policies and instructional strategies in gifted education.

Previous meta-analyses and review studies investigating the effects of enrichment programs on gifted children have consistently reported positive outcomes, particularly in the domain of academic achievement [8,22–24]. These findings suggest that enrichment interventions can enhance school performance, critical thinking, and creativity among gifted students. However, the impact of such programs on social-emotional variables—such as attitudes, self-concept, and interpersonal skills—remains less clear, with existing studies producing mixed results. However, no substantial effects were found on students’ self-concept, indicating that while enrichment supports cognitive and academic outcomes, its influence on self-perception and socio-emotional well-being may be limited. A more recent meta-analysis by Kim [8], which included 26 empirical studies published since 1985, further underscored the positive impact of enrichment on academic development. Kim’s meta-analysis found that enrichment programs yield positive effects on gifted students’ academic achievement and socioemotional development. Overall, the pooled effect sizes were large for academic outcomes (*g* = 0.96) and moderate for socioemotional outcomes (*g* = 0.55). Crucially, Kim examined grade level as a moderator and reported that effect sizes varied by grades. Academic effects of enrichment steadily increased across grade levels—moderate in elementary (*g* = 0.57), large in middle school (*g* = 1.37), and largest in high school (*g* = 2.02). In contrast, socioemotional benefits peaked in middle school (*g* = 0.93), were moderate at the elementary level (*g* = 0.43), and diminished by high school (*g* = 0.29). Thus, enrichment’s academic gains grow with grade, whereas its socioemotional advantages reach their zenith in early adolescence. Kim’s study applied strict inclusion criteria, selecting only those studies that clearly defined the enrichment programs used, provided sufficient quantitative data for effect size calculation, and employed either experimental or repeated-measures research designs.

As research on enrichment programs evolves and new interventions emerge, it is essential to systematically assess their effectiveness. Although Kim’s meta-analysis provided foundational insights into general academic and socioemotional outcomes, studies published between 2014 and 2024 exhibit considerable methodological heterogeneity: definitions of “enrichment” vary widely, outcome measures often rely on unvalidated instruments or small-scale designs, and moderator analyses exploring learner characteristics (e.g., prior achievement, cultural background) and delivery formats (e.g., hybrid vs. in-person) remain scarce. Furthermore, recent innovations—such as technology-enhanced and project-based models—have not been evaluated collectively in meta-analytic frameworks, and equity dimensions, including differential benefits across demographic groups, are largely unaddressed. In line with Borenstein’s [25] recommendation for periodic updates, a new synthesis that standardizes intervention and outcome coding, incorporates nuanced skill domains, and investigates critical moderators is therefore warranted to advance both theoretical understanding and practical application in gifted education.

### Gifted students

The concept of giftedness has undergone significant transformation, evolving from a narrow focus on high IQ to a more holistic and multidimensional understanding of talent and ability [26]. Traditional models primarily equated giftedness with elevated intelligence quotient scores; however, contemporary perspectives now encompass a broader set of indicators reflecting its complexity, including creativity, motivation, and domain-specific strengths [27]. Researchers such as Gagne [28] –relatively traditional- have expanded the definition of giftedness to include exceptional performance or potential in a wide range of domains such as science, technology, engineering, arts, mathematics (STEAM), leadership, and sports. This shift highlights the importance of recognizing giftedness not solely through general intellectual ability, but also through specialized talents that require differentiated identification methods and educational interventions. Gifted children are thus viewed as a diverse population exhibiting high potential across cognitive, linguistic, and interpersonal dimensions [29]. Multidimensional models, including Renzulli’s three-ring model [30], define giftedness as the interaction of above-average ability, creativity, and task commitment. In line with this, the National Association for Gifted Children [31] defines gifted students as those who perform, or have the potential to perform, at levels significantly above their peers in one or more domains. Moreover, various conceptual frameworks underscore the developmental nature of giftedness, suggesting that domain-specific skills can emerge and evolve over time, influenced by both internal dispositions and external factors such as family support, peer environment, and educational opportunities [32,33].

### Enrichment programs

Enrichment programs are widely recognized as essential educational strategies for meeting the advanced needs of gifted students. Although the term is frequently used in gifted education literature, it often lacks a universally accepted definition. Broadly, enrichment refers to instructional approaches that go beyond the standard curriculum, offering students opportunities to explore subjects in greater depth and complexity [12,34,35]. These programs are specifically designed to cultivate higher-order thinking, emotional intelligence, research competencies, communication skills, and creative problem-solving abilities [16,34]. The ultimate goal is to support both intellectual and personal growth by engaging students in meaningful learning experiences that challenge their capabilities and foster self-directed learning. Enrichment programs often emphasize deep content knowledge and advanced skill development within specific domains, aligning instructional content with students’ cognitive strengths and interests. These programs typically incorporate two core dimensions: content delivery and learning processes. Enrichment can be categorized as either vertical, which involves delving deeper into topics already covered in the curriculum, or horizontal, which introduces new and diverse subject matter beyond grade-level expectations [36]. Both types are designed to prevent underachievement, reduce disengagement, and provide intellectually stimulating opportunities that promote student motivation and perseverance [37]. Furthermore, enrichment initiatives often utilize flexible teaching strategies, such as inquiry-based learning, project-based tasks, mentorships, and interdisciplinary units, to engage gifted learners at higher cognitive levels [10]. For example, the Mawhiba-IAU program in Saudi Arabia demonstrates how well-designed enrichment initiatives can foster academic excellence, personal growth, and overall satisfaction among gifted students [18]. In essence, enrichment programs serve as dynamic and adaptive frameworks that support gifted students’ learning, thinking, and affective development by creating opportunities for both academic challenge and personal fulfillment.

### Cognitive skills

For gifted students, the development of robust cognitive skills is essential to realizing their full academic and personal potential. These skills serve as foundational tools that extend beyond specific content areas, enabling effective engagement with complex learning tasks across various domains [34]. One of the most important of these cognitive skills is the ability to learn. Learning skills are especially critical for gifted students, as they facilitate the organization, processing, and application of knowledge—allowing them to navigate and make the most of enriched educational opportunities. Sayı and Yurtseven [3] identify several core learning skills that are particularly relevant for gifted learners, including listening, observing, perceiving, reading comprehension, note-taking, outlining, data analysis and organization, and effective communication. Reis et al. [34] further emphasize that learning skills also encompass planning, organizing, resource utilization, time management, decision-making, and self-assessment. These competencies enable students to become autonomous learners capable of setting goals, monitoring their progress, and reflecting on their learning strategies. As Macleod and Hayden [38] suggest, educators play a crucial role in fostering these abilities by providing learning environments that promote active participation, strategic thinking, and independent inquiry. Given the advanced cognitive abilities and diverse learning needs of gifted students, the intentional development of these skills is not merely beneficial but necessary. Strengthening learning skills equips gifted students to meet academic demands more effectively, take intellectual risks, and pursue their interests with greater focus and resilience. Moreover, these competencies support long-term academic achievement and lifelong learning by fostering adaptability, critical self-reflection, and a proactive approach to problem-solving. As such, enrichment programs that target learning skill development contribute significantly to the comprehensive educational experience of gifted students.

In addition to advancing learning skills, enrichment programs are designed to promote thinking skills, which stand as another central facet of gifted students’ cognitive abilities. These skills are generally categorized into two main domains: creative thinking and analytical thinking [8,39]. Creativity is widely recognized as a hallmark of giftedness, and its development is a central component of instructional strategies tailored for gifted learners. Creative thinking involves approaching situations from novel perspectives and generating innovative solutions. Key components of creative ability include: (1) fluency—the capacity to produce a large number of ideas; (2) flexibility—the ability to shift perspectives or apply ideas across different contexts; (3) elaboration—the skill of expanding on ideas with detail; and (4) originality—the ability to generate unique and unconventional responses. These elements collectively enable students to engage in divergent thinking, which is essential for innovation and problem-solving in complex, real-world situations. In parallel, analytical thinking encompasses skills related to information processing and logical reasoning. These include comparing and contrasting, identifying relationships, classifying, ordering, and interpreting data [20]. Analytical thinking enables students to make inferences, recognize patterns, and evaluate evidence systematically—abilities that are crucial for both academic performance and the development of scientific reasoning. Enrichment programs designed for gifted students frequently incorporate tasks that promote these skills, such as structured problem-solving, hypothetical reasoning, and critical analysis of arguments. Moreover, many enrichment models integrate research-based competencies into cognitive skill development. These include the ability to define problems, collect and analyze data, observe systematically, and use resources effectively [40]. Through the development of both creative and analytical thinking, gifted students can approach learning tasks with depth, originality, and independence. Enrichment interventions that foster these cognitive abilities contribute not only to immediate academic success but also to the cultivation of advanced intellectual potential.

### Non-cognitive skills

#### Personal affective skills.

Personal affective skills are critical for the holistic development of gifted students, as they influence motivation, self-regulation, and emotional well-being—factors that directly affect both academic performance and long-term success [32]. These skills encompass a range of intra-individual competencies, including self-awareness, self-confidence, emotional regulation, and personal goal-setting. While gifted students often demonstrate advanced cognitive abilities, their affective development may not progress at the same pace, and thus requires intentional support through educational interventions [41]. Enrichment programs are increasingly recognized for their potential to nurture these aspects of development. As Subotnik et al. [32] emphasize, socioemotional factors play a vital role in the realization of gifted students’ potential. A well-designed enrichment program addresses not only intellectual needs but also supports affective growth by fostering a positive self-concept, emotional resilience, and autonomy [20]. Self-concept—one’s perception of self in academic and social contexts—is a foundational component of personal affective development. It includes several dimensions. *Self-esteem*, or the overall value individuals assign to themselves, which tends to remain relatively stable over time [42]. *Academic self-concept*, which reflects students’ beliefs about their abilities in specific academic domains [8]. *Social self-concept*, which refers to perceived competence and acceptance in social interactions [43]. Closely related to self-concept is self-efficacy, which refers to individuals’ confidence in their ability to successfully perform specific tasks or achieve goals [44]. High self-efficacy is associated with persistence, resilience, and adaptive learning strategies—all of which are crucial for gifted students navigating challenging academic and personal environments. Another vital dimension is self-management, which involves organizing and regulating one’s behavior to achieve learning goals. This includes emotional self-control, integrity, optimism, adaptability, achievement orientation, and initiative [45]. Motivation is also a central driver of gifted students’ engagement and performance; it sustains their interest in challenging tasks and enables them to seek out enriching experiences with like-minded peers. In summary, personal affective skills—including self-concept, self-efficacy, motivation, and self-management—are essential for gifted students’ overall development. Enrichment programs that address these areas provide crucial support for their emotional and psychological needs, enabling them to thrive both academically and personally.

#### Social affective skills.

While giftedness is often associated with advanced intellectual abilities, it does not inherently guarantee strong social skills. In fact, many gifted students experience challenges in social development due to factors such as asynchronous growth, perfectionism, heightened sensitivity, or a sense of isolation from age peers [46]. These challenges can hinder their ability to engage effectively in interpersonal relationships and collaborative environments. Therefore, enrichment programs that intentionally support social development play a crucial role in helping gifted learners build essential interpersonal competencies. Social affective skills, also referred to as interpersonal or relational skills, are vital for success in both academic and personal contexts. These include the ability to navigate social situations, communicate effectively, collaborate with others, and demonstrate empathy and leadership [19]. Developing these skills enables gifted students to form meaningful relationships, contribute to group efforts, and adapt to diverse social environments. They also support broader educational outcomes by fostering emotional intelligence and social adaptability.

A key component of social competence is social awareness, which involves recognizing and appropriately responding to the emotions, perspectives, and needs of others. According to Boyatzis [45], social awareness comprises skills such as empathy, organizational awareness, and service orientation—each of which enhances students’ ability to function effectively in group settings. Building on this, Yudha and Ropipah [47] introduce the concept of relationship management, a broader framework that includes guiding group processes, managing conflicts, inspiring and motivating others, and fostering cooperative dynamics. These skills are especially valuable in project-based enrichment activities, leadership development programs, and collaborative problem-solving scenarios. Social competence, as described by Rose-Krasnor [48], refers to the ability to achieve personal goals in social interactions while maintaining positive relationships over time and across different contexts. For gifted students, strengthening social affective skills is not only important for their psychological well-being but also for their participation in complex, team-oriented, and interdisciplinary environments often emphasized in enrichment programs. In conclusion, enrichment programs that incorporate components focused on social development provide gifted students with the tools they need to enhance their interpersonal functioning. These skills contribute to their overall adjustment, emotional health, and capacity to lead and collaborate—preparing them for success both within and beyond academic settings.

### Research aim and research questions

This study aims to systematically evaluate the effectiveness of enrichment programs on the development of gifted students’ skills across three key domains: cognitive skills, affective personal skills, and affective social skills. Unlike prior meta-analyses—such as Kim [6], which focused on broader constructs like academic achievement and general socioemotional development—this study adopts a more targeted and nuanced approach by disaggregating the outcomes into distinct cognitive and affective subdomains. This allows for a clearer understanding of how specific enrichment strategies influence diverse aspects of gifted students’ development. The originality of this research lies in both its scope and precision. First, it is the most up-to-date meta-analysis conducted on enrichment programs for gifted students, incorporating empirical studies published between 2014 and 2024—a period marked by significant educational shifts, including the global adaptation to remote learning during the COVID-19 pandemic. Second, it provides a domain-specific evaluation, offering separate effect size estimates for cognitive, affective personal, and affective social skills. This fine-grained analysis moves beyond general measures of “achievement” or “development” and responds directly to the call in the literature for more differentiated and actionable findings [25]. By updating and extending previous meta-analytic findings, this study offers both theoretical contributions—clarifying the distinct pathways through which enrichment impacts various skill sets—and practical implications for curriculum designers, educators, and policymakers who aim to implement evidence-based enrichment strategies tailored to gifted learners. Based on the objectives outlined above, the research questions guiding this study are presented below:

1. What is the overall effect of enrichment programs on the cognitive skills of gifted students?
2. What is the overall effect of enrichment programs on the non-cognitive skills (affective personal and affective social) skills of gifted students?
3. How do different program types influence the effects of enrichment programs on both cognitive and affective skills?
4. How do different grade levels influence the effects of enrichment programs on both cognitive and affective skills?

These questions are designed to explore the specific contributions of enrichment interventions across cognitive and emotional dimensions, providing a clearer evidence base for optimizing gifted education practices.

## Methods

A meta-analysis method was employed to examine the effects of enrichment programs on the development of cognitive, and affective personal and social skills in gifted students, based on experimental studies. Borenstein [25] stated that meta-analysis is a statistical analysis which provides combining the results of multiple individual studies on the same subject systematically. As described by Cohen [49], meta-analysis inherently requires synthesizing a large amount of quantitative data in a consistent way that take effect sizes into account in order to draw meaningful conclusions by systematically analysing these data.

The review was conducted through a systematic review and meta-analysis of existing research on the effects of enrichment on gifted students’ cognitive, and affective personal and social skills. To conduct a systematic review, the PRISMA statement and the practical guide for systematic reviews were used [50]. During the screening process, we employed some criteria for the inclusion and exclusion of studies. Studies were included if: (1) published between 2014 and 2024 (until 31 December 2024), (2) involving participants between grades 1 and 12, (3) using enrichment intervention to investigate the impact of enrichment programs (ER) on the learning, thinking and affective abilities of gifted students in the experimental group, (4) using traditional interventions in the control group, (5) quantitative studies that effectively reported statistical data to calculate effect sizes, and (6) Turkish and English studies. Studies were excluded if: (1) involving grouping or any acceleration intervention, (2) involving participants attending university, (3) involving a control group participating outside of traditional teaching, (4) not reporting quantitative results, (5) not available in full text, (6) conference proceedings, editorials and reviews.

Searches were carried out Web of Science, and Scopus databases for publications. Eligible studies were published from 2014 to 2024. Two sets of keywords were used in research query. The former included gifted, talented, highly able, highly achieve, high performing, and genius. The latter consisted of enrichment, extracurricular, pull-out program, summer program, talent development, separate class, camp, science camp, math camp, technology camp, art camp, sport camp. These two sets of keywords were combined with the Boolean operators (AND, OR). The research query was applied to above databases by keying in title, abstract, and keywords. The research strings were as follows: TS=(“gifted” OR “talent*” OR “highly able” OR “highly achieve” OR “high perform*” OR “genius” OR “intelligent”) AND TS=(enrichment OR extracurricular OR pull-out program OR summer program OR talent development OR separate class). After implementing the research query, the refinement process was conducted by selecting the categories of Education & Educational Research, Education Special, Education Scientific Disciplines, and Educational Psychology within the WoS for the period 2014–2024. The selection was limited to the SSCI, SCI-Expanded, ESCI, and A&HCI indexes.

**Fig 1:**
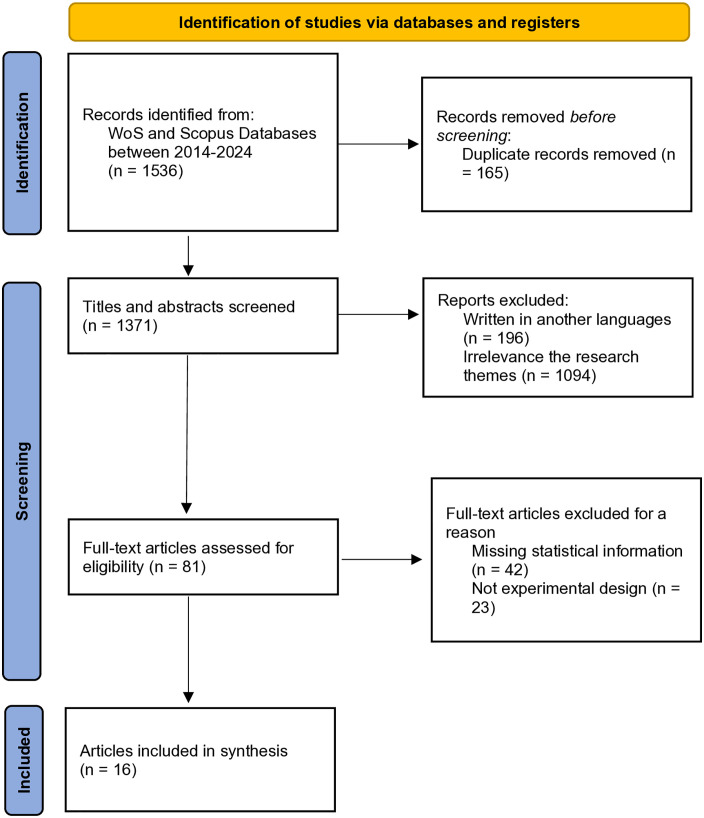
Research review diagram.

The coding procedure was conducted by both authors to assess eligibility based on the types of enrichment programs, number of students, grade level or age, types of comparison, and outcome measures. The inter-rater reliability was 0.93. Disagreement on the quality assessment was discussed and resolved by two authors. After the screening process, study selection was carried out. A total of 1536 studies were identified and 165 studies were excluded due to duplication. The removal of duplicate studies was performed using the RStudio software by executing the following code: [(library(bibliometrix), A = convert2df(“wos.bib”, dbsource = “wos”, format = “bibtex”), B = convert2df(“scopus.bib”, dbsource = “scopus”, format = “bibtex”), C = mergeDbSources(A,B, remove.duplicated = T))}. Following the initial retrieval, titles and abstracts were screened, resulting in the exclusion of 196 studies due to being written in a non-target language and 1094 studies for not aligning with the research themes. Then, out of 81 studies, 65 studies were excluded because they did not meet the following inclusion criteria: (1) lack of sufficient statistical data to measure effect size, (2) not focusing on enrichment intervention through experimental and quasi-experimental studies. As a result, a total of 16 studies were considered for meta-analysis (Fig 1).

### Data analysis

Comprehensive Meta-Analysis software (3. version) (CMA) was used to carry out the meta-analysis in a step-by-step manner. A random effect model and a 95% confidence interval to calculate the pooled effect size was employed, since significant heterogeneity could not be eliminated because of possible variations in the designs of enrichment models and study contexts.

Hedges’s *g* was used to report the magnitude or strength of the differences between groups in a study as effect size, since it provides modifying effect size for smaller groups [51,52].

**Fig 2:**
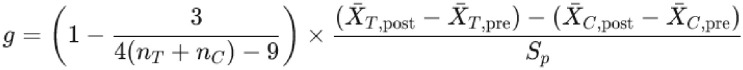
Hedges’s *g* Formula.

Hedges’s *g* enables reporting effect sizes in terms of standard deviation units that provides making comparison between groups (see formula in Fig 2). As stated by Cohen [51], a Hedges’s *g* value below 0.5 is considered to represent a small effect, while 0.50 to 0.79 is considered to indicate a medium effect, and a value of 0.80 or higher is classified as a large effect. Meta-analyses were performed separately for studies focusing on learning, research, thinking, and affective skills.

*Publication bias* was reported to address the concern that the current literature may not enable a comprehensive representation of the research carried out in this field [52]. Biased results weaken the validity of the meta-analysis [53]. The researchers examined publication bias concerning learning outcomes by analysing data using various methods, including the funnel plot, Begg’s test, Egger’s test, and trim-and-fill test. Additionally, they utilized Classic fail-safe N and Orwin’s fail-safe N values for further analysis.

*Heterogeneity* was reported to assess the likelihood that the variance in effect sizes observed could be attributed to sampling error solely [54]. To examine the variation in effect sizes, the *Q*-statistic [52] and the *I*^*2*^-statistic [55] were employed. As pointed out by Cooper et al. [54] and Ellis [56], if the *Q* statistic surpasses the critical value, that means the factor contributes the variance in effect size significantly, thereby the hypothesis of homogeneity is rejected. This indicates that the population of effect size is heterogeneous and requires that diverse effects have been detected for various groups concerning moderator variables. According to *I*^*2*^ values, while *I*^*2*^ ≤ 50% indicates low heterogeneity, 50 ≤ *I*^*2*^ ≤ 75 shows medium heterogeneity, and values other than these ranges were considered high heterogeneity. When this value is higher than 75%, the random effect model is more suitable for the meta-analysis, otherwise the fixed effects model is required [25].

## Results

All 16 studies included in the research were published as journal articles. Although some studies do not directly focus on specific grade levels, and since some studies were conducted in more than one group, it is revealed that the majority of the studies were at the primary (11 studies, grades 1–6) and middle school level (7 studies, grades 10–12). The rest were conducted in middle school level (4 studies, grades 7–9). Enrichment studies conducted in primary school settings predominantly focused on affective and cognitive abilities. In contrast, middle school studies cantered on learning and affective competencies, while high school studies addressed learning, cognitive, and affective skills.

In terms of research designs employed by the studies, the vast majority of them (n = 13) were quasi-experimental design. The treatment groups in all these studies were enrolled in different types of enrichment programs, such as summer, Saturday, extracurricular, school wide, and state wide enrichment programs. Table 1 provides an overview the descriptive information of the studies including author, year, design, sample, intervention, and outcome measure.

**Table 1:** Studies on enrichment interventions.

Author	Types of comparison	Participants		Enrichment program	Outcome measure
		N		Grade level	
Alkhuzaim and Al-Qutaim [15]	One group pre-post quasi experimental design	42	9-10	Inquiry based enrichment program (Academic year)	- Innovative thinking, Curiosity, Perseverance, Imagination, Collaboration
Ayoub et al. [57]	One group pre-post quasi experimental design	60	8-9	Summer enrichment program (Summer-day)	- Belief identification, Dogmatic thinking, Flexible thinking, Open-mindedness, Problem finding
Batterjee [16]	Non-equivalent groups pre-post quasi experimental design	C = 267 T = 228	4-11	Pull-out and separate class program (Academic year)	- Self-perception, Learning
Darga and Ataman [58]	One-group pre-post test experimental design	31	1	Class-wide enrichment program (Academic year)	Achievement
Elhoweris et al. [59]	Pre-post test experimental design	C = 20 T = 20	4	Reading enrichment program (Academic year)	Critical reading
Foley-Nicpon et al. [60]	Non-equivalent group pre-post quasi experimental design	C = 9 T = 28	4-5	Summer enrichment program (Summer-day)	- Closeness, Companionship, Conflict, Help, Security
García-Perales and Almeida [17]	Non-equivalent groups pre-post quasi experimental design	C1 = 27 C2 = 9 T = 9	2-6	Summer enrichment program (Summer-day)	- School maladaptation
Golle et al. [10]	Non-equivalent groups pre-post quasi experimental design	C = 2328 T = 423	2-4	A state-wide extracurricular enrichment program (Academic year)	- Creativity, Crystalize intelligence, Curiosity, Self-concept, Social competence
Gubbels et al. [19]	Pretest-posttest experimental design	C = 20 T = 26	4	Computer based enrichment programme (Academic year)	- Analytical ability, Creative ability
Muammar [18]	One group pre-post quasi experimental design	669	9-12	An innovative gifted summer program (Summer-day)	- Achievement, Creativity, Entrepreneurship, Leadership, Problem solving
Mun and Hertzog [61]	One group pre-post quasi experimental design	40	4-8	Saturday enrichment program (Academic year)	- Math attitude, Math identity
Preckel et al. [41]	One group pre-post quasi experimental design	177	10-12	Summer day program	- Academic self-concept, Social self-concept
van Rossen et al. [62]	Non-equivalent groups pre-post quasi experimental design	C = 429 T = 245	4-6	Pull-out and separate class program (Academic year)	- Social relationship
Vidergor [63]	Non-equivalent groups pre-post quasi experimental design	C = 195 T = 199	4-8	Extracurricular enrichment program (Academic year)	- Scientific thinking, Creative thinking, Future thinking
Vidergor et al., [64]	Non-equivalent groups pre-post quasi experimental design	C = 168 T = 166	4-8	Extracurricular enrichment program (Academic year)	- Future thinking
Yoon et al. [65]	One group pre-post quasi experimental design	14	9-12	Youth science and technology leadership camp (Summer day)	- Self-awareness, Self-confidence, Learning

### Publication bias

The meta-analysis comprised 16 studies, with a total of 44 effect sizes under examination. In line with the methodology outlined, we applied several strategies to identify and mitigate the risk of publication bias. These included scrutinizing funnel plots, performing Begg’s Test, and conducting Egger’s Regression Test and the classic fail-safe N Analysis, implementing Duval and Tweedie’s Trim and Fill Analysis, It’s noteworthy that publication bias stemming from various origins could potentially influence the findings of meta-analytic studies. Notably, the publication of exclusively positive study results is a significant contributor to this bias [66].

**Fig 3:**
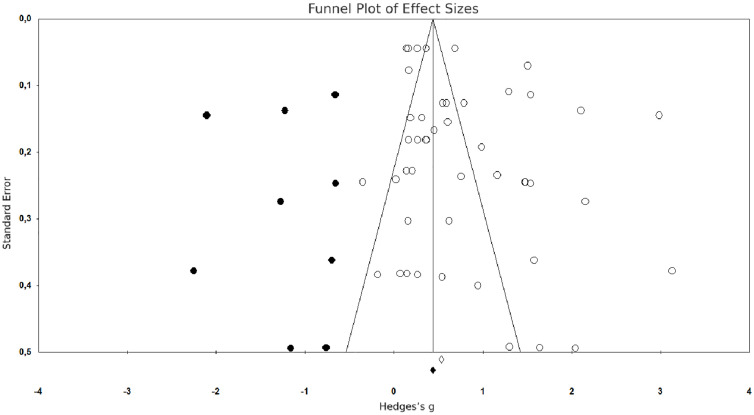
Funnel plot of standard error by Hedges’s g.

Assessing the validity of meta-analyses can be facilitated by funnel plots. These plots illustrate included studies with empty circles, while studies necessary to eradicate bias are represented by full circles [51]. In instances of publication bias within meta-analyses, funnel plots take on an asymmetrical form, indicating that both bias and genuine heterogeneity of potential effects contribute to this observation Egger et al. [67]. Notably, the funnel plot depicted in Fig 3 displayed asymmetry, suggesting the potential presence of publication bias.

In terms of research designs employed by the studies, all studies were quasi-experimental design. The treatment groups in all these studies were enrolled in different types of enrichment programs, such as summer, Saturday, extracurricular, school wide, and state wide enrichment programs. Given the subjective nature of interpreting the funnel plot as an informal means of assessing publication bias, the Begg’s and Egger regression tests were employed for further evaluation. Begg’s test, alternatively referred to as the rank correlation test, assesses the correlation between the standardized effect and effect variance by employing Kendall’s Tau rank correlation test, specifically examining the relationship between the test effect and sample size. In the Begg and Mazumdar rank correlation, which incorporates Kendall’s Tau with a continuity correction, the outcomes (*Z* = 1.153 < 1.96, *p* = 0.25 > 0.05) suggest the presence of insignificant publication bias [68].

Egger’s test evaluates the association between the observed effect size and the standard error. The obtained *p*-value from this test suggested the absence of publication bias (*p* = .07 > .05). Egger’s test indicates an absence of publication bias in terms of primary education (*z* = 0.46, *p* = 0.724) and secondary education (*z* = −2.34, *p* = 0.101) but indicates a presence of publication bias in terms of higher education (*z* = 4.84, *p* < 0.001) and overall educational levels (*z* = 4.37, *p* < 0.001).

In addition, Duval and Tweedie’s trim-and- fill technique was employed to test publication bias and obtain an unbiased estimate of the effect size [66]. The trim-and-fill technique recommended that nine hypothetical missing studies should be added in order to obtain a symmetric funnel plot (Fig 3). It indicates a presence of publication bias, possibly due to the influence of small study bias on learning outcomes. The imputed effect size was 0.820 (95% CI = [0.640, 1.000]), indicating a significantly high effect of enrichment studies on overall effect (*p* < 0.001). Rosenthal’s fail-safe N was 2,705, indicating that 2,705 null-result studies would be required to reduce the combined effect to non-significance at α = .05. This value surpasses the 205 threshold obtained through the formula 5k + 10, as suggested by Fragkos et al. [69]. Based on all these findings, we conclude the absence of publication bias.

### Overall effect size and heterogeneity test

In terms of research designs employed by the studies, all studies were quasi-experimental design. The treatment groups in all these studies were enrolled in different types of enrichment programs, such as summer, Saturday, extracurricular, school wide, and state wide enrichment programs. The relationship between enrichment programs and the overall effect of skills of students is presented in Table 2 below. Regarding evaluation criteria according to Cohen [51], combined effect size of enrichment studies on students’ skills (*g* = 0.820, 95% CI: 0.640, 1.000, *Z* = 8.875, *p* < 0.001) was higher than.80, indicating that enrichment studies had large effect on students’ skills.

**Table 2:** Overall effect sizes and the heterogeneity tests of enrichment studies (random-effects model).

Statistics	Values
*k*	16
*N*_*es*_	44
Hedges’ *g* [CI (95%)]	.820 [0.640, 1.000]
Tau-squared	.320
*p*	.000
*z-value*	8.875
Classic fail safe-*N*	2705
Trim & fill	9
**Heterogeneity**	
Q_*total*_	1173.08
*p-value*	.000
*df*	43
*I* ^*2*^	96.334%

The *Q*-test outcomes indicated a noteworthy variation in effect sizes among studies (*Q* = 1173.08; df = 43, *p* < 0.001). Additionally, the *I*^*2*^ statistic was determined to be 96.33, signifying substantial heterogeneity in effect sizes. Specifically, approximately 96.33% of the observed variance discrepancy can be attributed to a genuine effect rather than sampling error. Consequently, the random-effect model was employed to address sample heterogeneity and ensure the robustness of the scientific analysis results.

**Fig 4:**
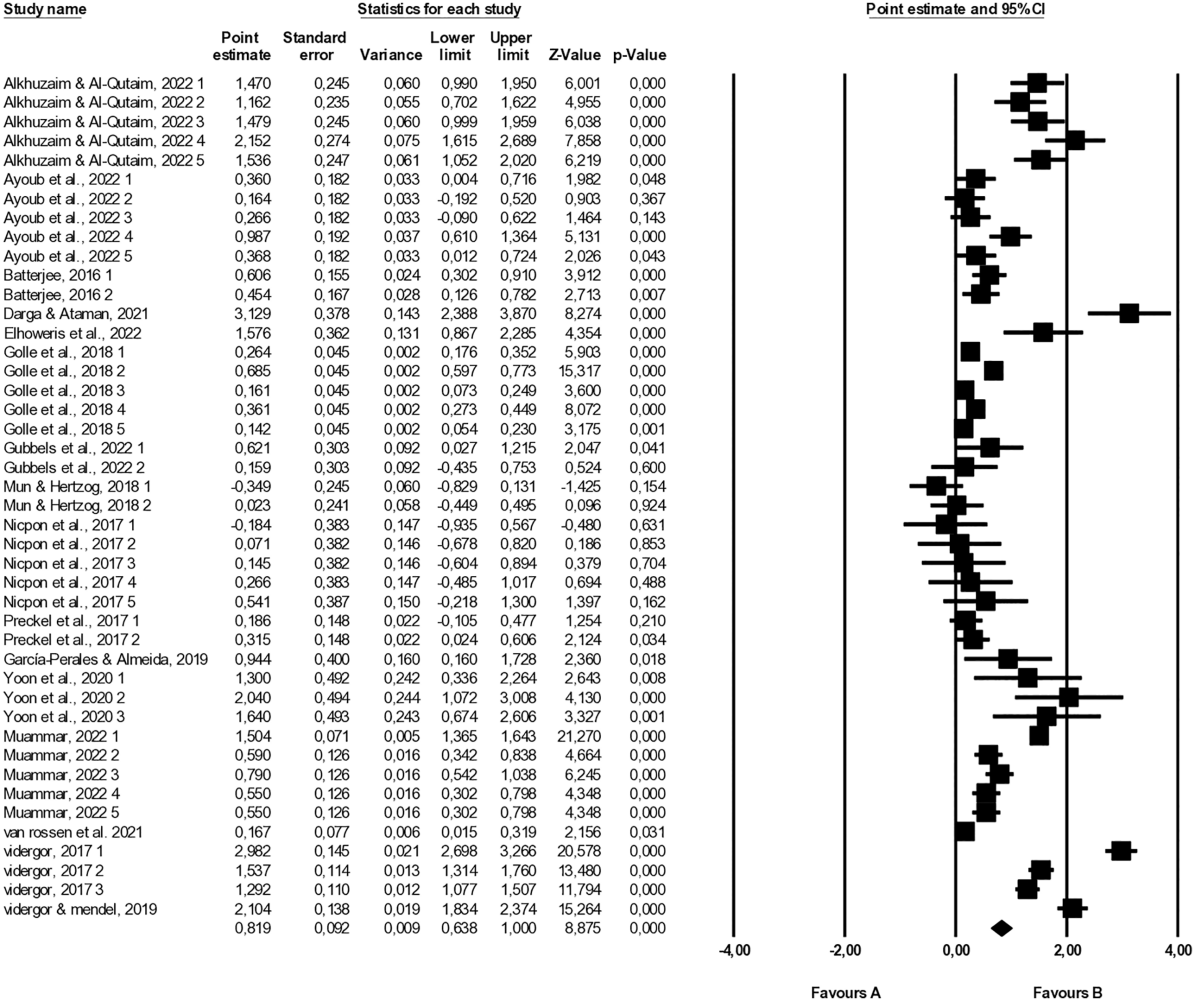
Forest plot of effect sizes using the random effects model.

Random-effects model was used in order to pool the effect sizes. The random‐effects model in meta‐analysis is designed to pool study results while explicitly accounting for between‐study variation (heterogeneity) in true effects. The random-effects model in meta‐analysis assumes that each study’s true effect varies around an overall mean μ, rather than being identical as in a fixed‐effect model. In 44 effect size were extracted from 16 studies regarding cognitive (21 effect sizes), and affective –social (12 effect sizes) and personal (11 effect sizes) – skills. Table 2 and Fig 4 displays the effect sizes represented as Hedge’s g for each dimension, alongside pertinent statistical details such as standard error, the lower and upper limits of the 95% confidence interval (CI) for the effect size, *Z* value, and corresponding *p* value.

#### Cognitive skills.

Among the included studies, twenty one (21) effect sizes were extracted for the impact of enrichment studies on cognitive skills of gifted students. The random-effects model demonstrated that the effect sizes ranged from 0.16 to 3.13. A large effect was found on the mean effect size (*g* = 1.14, 95% CI [.83, 1.45]), indicating that enrichment interventions had a large effect on thinking skills (Table 3). In light of the varied variance across studies, the *Q* value was determined to be 742.34 (df = 20; *p* < .001), thereby refuting the null hypothesis positing identical true effect sizes across studies. Moreover, the *I*^*2*^ statistic of 97.31 signifies substantial heterogeneity in effect sizes among the studies. This statistic suggests that approximately 97.31% of the observed variance in effect sizes can be attributed to genuine differences rather than sampling error.

**Table 3:** Effectiveness of enrichment on cognitive skills.

	Study	Effect sizes	SE	Lower limit	Upper limit	*Z*	*p*
1	Alkhuzaim and Al-Qutaim [15]	1.48	0,25	0,99	1,95	6,03	0.00
	Alkhuzaim and Al-Qutaim [15]	2.15	0,27	1,61	2,69	7,85	0.00
2	Batterjee [16]	0.61	0.16	0.30	0.91	3.91	0.00
3	Ayoub et al. [57]	0.16	0,18	−0,19	0,52	0,90	0.37
	Ayoub et al. [57]	0.27	0,18	−9,00	0,62	1,46	0.14
	Ayoub et al. [57]	0.99	0,19	0,60	1,36	5,13	0.00
	Ayoub et al. [57]	0.37	0,18	1,19	0,72	2,02	0.04
	Darga and Ataman [58]	3.13	0.38	2.39	3.87	8.27	0.00
4	Elhoweris et al. [59]	1.58	0,36	0,86	2,28	4,35	0.00
5	Golle et al. [12]	0.26	4,47	0,17	0,35	5,90	0.00
	Golle et al. [12]	0.68	4,47	0,59	0,77	15,31	0.00
6	Gubbels et al. [19]	0.62	0,30	2,65	1,21	2,04	0.04
	Gubbels et al. [19]	0.15	0,30	−0,43	0,75	0,52	0.60
7	Muammar [18]	0.59	0,12	0,34	0,83	4,66	0.00
	Muammar [18]	0.55	0,12	0,30	0,79	4,34	0.00
	Muammar [18]	1.50	0.07	1.37	1.64	21.27	0.00
8	Vidergor [63]	2.98	0,15	2,70	3,27	20,58	0.00
	Vidergor [63]	1.54	0,11	0,01	1,31	13.48	0.00
	Vidergor [63]	1.30	0,11	0,01	1,08	11,80	0.00
9	Vidergor et al. [64]	2.10	0,14	1,83	2,37	15,26	0.00
10	Yoon et al. [65]	1.30	0.49	0.34	2.26	2.64	0.00
	Total	1.14	0,16	0,83	1,45	7,17	0.00

#### Affective personal skills.

Among the included studies, eleven (11) effect sizes were extracted for the impact of enrichment studies on affective personal skills of gifted students. The random-effects model demonstrated that the effect sizes ranged from −0.35 to 2.04. A medium effect size was found on the mean effect size (*g* = .51, 95% CI [.30,.73]), indicating that enrichment interventions had a moderate effect on affective personal skills (Table 4).

**Table 4:** Effectiveness of enrichment on affective personal skills.

	Study	Effect sizes	SE	Lower limit	Upper limit	*Z*	*p*
1	Alkhuzaim and Al-Qutaim [15]	1,16	0,24	0,70	1,62	4,96	0.00
	Alkhuzaim and Al-Qutaim [15]	1,54	0,25	1.05	2,02	6,22	0.00
3	Ayoub et al. [57]	0,36	0,18	0,00	0,72	1,98	0.04
4	Batterjee [16]	0,45	0,17	0,13	0.78	2,71	0.00
5	Golle et al. [12]	0,16	0,05	0,07	0.25	3,60	0.00
	Golle et al. [12]	0,36	0,05	0,27	0.45	8,07	0.00
6	Mun and Hertzog [61]	−0,35	0,25	−0.83	0.13	−1,43	0.15
	Mun anf Hertzog [61]	0,02	0,24	−0,45	0.50	0,10	0.92
7	Preckel et al. [41]	0,19	0,15	0,47	−0.11	1,25	0.21
8	Yoon et al. [65]	2,04	0,49	3,00	3.01	4,13	0.00
	Yoon et al. [65]	1,64	0,49	0,67	2.61	3,33	0.00
	Total	0.51	0.11	0.30	0.73	4.58	0.00

Concerning the diverse variability observed across studies, the *Q* value obtained is 79.75 (df = 10; *p* < .001), leading to the rejection of the null hypothesis which assumes identical true effect sizes across studies. Furthermore, the *I*^*2*^ statistic of 87.46 indicates substantial heterogeneity in effect sizes among the studies. This statistic suggests that approximately 87.46% of the observed variance in effect sizes can be attributed to genuine differences rather than sampling error.

#### Affective social skills.

Among the included studies, twelwe (12) effect sizes were extracted for the impact of enrichment studies on affective social skills of gifted students. The random-effects model demonstrated that the effect sizes ranged from −0.18 to 1.47. A small effect was found on the mean effect size (*g* = .44, 95% CI [.23,.66]), indicating that enrichment interventions had a small effect on affective social skills (Table 5).

**Table 5:** Effectiveness of enrichment on affective social skills.

	Study	Effect sizes	SE	Lower limit	Upper limit	*Z*	*p*
1	Alkhuzaim and Al-Qutaim [15]	1.47	0.25	0.99	1.95	6.00	0.00
3	Foley-Nicpon et al. [60]	−0.18	0.38	−0.94	0.57	−0.48	0.63
	Foley-Nicpon et al. [60]	0.07	0.38	−0.68	0.82	0.19	0.85
	Foley-Nicpon et al. [60]	0.14	0.38	−0.61	0.90	0.38	0.70
	Foley-Nicpon et al. [60]	0.27	0.38	−0.49	1.02	0.69	0.49
	Foley-Nicpon et al. [60]	0.54	0.39	−0.21	1.30	1.40	0.16
4	García-Perales and Almeida [17]	0.94	0.40	0.16	1.73	2.36	0.02
5	Golle et al. [12]	0.14	0.05	0.05	0.23	3.18	0.00
6	Muammar [18]	0.79	0.13	0.54	1.04	6.25	0.00
	Muammar [18]	0.55	0.13	0.30	0.80	4.35	0.00
7	Preckel et al. [41]	0.32	0.15	0.02	0.61	2.12	0.03
8	Van Rossen et al., [62]	0.17	0.08	0.02	0.32	2.16	0.00
	Total	0.44	0.11	0.23	0.66	4.03	0.00

Heterogeneity analysis revealed statistically significant variability in the true effect sizes across studies. The *Q*-statistic of 60.88 (df = 11; *p* < .001) decisively rejects the null hypothesis of homogeneity, while the I² statistic of 81.93 further supports this conclusion. This indicates substantial true heterogeneity (80.31%) beyond mere sampling error, warranting exploration of potential moderators or subgroup analyses.

#### Moderator analysis.

**Moderators of Effect Sizes for Studies of Enrichment Programs for Cognitive Skills.** For the moderator of types of program, the effect size of academic year and summer day programs were significantly different from zero, with effect sizes of 1.41 and.70. The *Q* value was statistically significant, indicating that types of programs influenced effect sizes. (Table 6). A large effect size was observed for academic year, and a middle effect size was observed for summer day program. For the moderator types of grade level, the effect size of grade levels were significantly different from zero, with effect sizes of 1.34, 1.10, and 1.14. The *Q* value was statistically significant, indicating that grade levels impacted effect sizes. A large effect size was observed for all grade levels.

**Table 6:** Moderators of Effect Sizes for Studies of Enrichment Programs for Thinking Skills.

Moderator	*k*	*Q*	Effect size	Lower limit	Upper limit	*p*
**Types of program**		609.08				
Academic year program	13		1.41	.97	1.85	.000
Summer day program	8		0.70	.26	1.13	.000
**Grade level**		575.47				
Primary level	11		1.34	.87	1.82	.000
Middle	13		1.10	.69	1.51	.000
High	7		1.14	.68	1.59	.000

**Moderators of Effect Sizes for Studies of Enrichment Programs for Affective Personal Skills.** For the moderator of types of program, the effect size of academic year and summer day programs were significantly different from zero, with effect sizes of.44 and.88. The *Q* value was statistically significant, indicating that types of programs influenced effect sizes. (Table 7). A small effect size was observed for academic year, and a large effect size was observed for summer day program. For the moderator types of grade level, the effect size of grade levels were significantly different from zero, with effect sizes of.21,.54, and 1.07. The *Q* value was statistically significant, indicating that grade levels impacted effect sizes. A small, middle, and large effect sizes were observed for primary, middle, and high levels respectively.

**Table 7:** Moderators of Effect sizes for studies of enrichment programs for affective personal skills.

Moderator	*k*	*Q*	Effect size	Lower limit	Upper limit	*p*
**Types of program**		59.11				
Academic year program	7		.44	.20	.70	.000
Summer day program	4		.88	.22	1.54	.000
**Grade level**		79.75				
Primary level	5		.21	.03	.39	.001
Middle	6		.54	.05	1.04	.000
High	6		1.07	.52	1.63	.000

**Moderators of Effect Sizes for Studies of Enrichment Programs for Affective Social Skills.** For the moderator of types of program, the effect size of academic year and summer day programs were significantly different from zero, with effect sizes of.47 and.46. The *Q* value was statistically significant, indicating that types of programs influenced effect sizes. (Table 8). Small effect sizes were observed for both academic year and summer day program. For the moderator types of grade level, the effect size of grade levels were significantly different from zero, with effect sizes of.16,.67, and.74. The *Q* value was statistically significant, indicating that grade levels impacted effect sizes. A small effect size was observed for primary level. Middle effect sizes were observed for middle and high levels.

**Table 8:** Moderators of effect sizes for studies of enrichment programs for affective social skills.

Moderator	*k*	*Q*	Effect size	Lower limit	Upper limit	*p*
**Types of program**		60.88				
Academic year program	3		.47	.10	.85	.000
Summer day program	9		.46	.25	.67	.000
**Grade level**						
Primary level	8		.16	.08	.23	.000
Middle	2		.67	.44	.91	.000
High	4		.74	.37	1.11	.000

## Discussion

This meta‐analysis addresses four principal questions concerning the effects of enrichment programs on various domains of gifted learners’ development. First, we estimated the overall effect size of enrichment interventions on cognitive skills among gifted students. According to the results, the observed effect size (*g* = 1.14) constitutes a large effect, indicating that enrichment programs substantially enhance gifted students’ cognitive skills. This robust positive outcome aligns with prior systematic reviews [70] and exceeds slightly the effect size reported by Kim (*g* = 1.06). This result also concurs with an earlier meta‐analysis [71], though it is slightly higher than the effect size reported by Lo and Feng (*g* = 0.76). While Kim [6] did not conduct a discrete analysis of cognitive skills, substantial effect sizes were noted for achievement‐related competencies that substantially overlap with cognitive‐process skills. Vaughn et al. [24] similarly documented positive outcomes for critical and creative thinking following enrichment. The emphasis within these programs on scientific inquiry processes—such as problem identification, deduction, induction, and evaluation—further supports their efficacy in developing analytical abilities, as corroborated by Alkhuzaim and Al-Quatim [15]. Additional pedagogical strategies, including presenting content in abstract and interdisciplinary contexts [56,59], engaging students with ill‐defined problems [19], and providing explicit instruction targeting higher‐order skills, have been shown to amplify these effects. Golle et al. [12] and Gubbels et al. [19] further argue that diverse, interest‐driven course offerings and personalized learning environments foster intrinsic motivation and peer‐mediated challenges, thereby stimulating critical thinking and independent inquiry.

Although empirical research on enrichment has grown in recent years [71], truly experimental investigations remain scarce. Our findings corroborate earlier evidence regarding the benefits of acceleration [72] but diverge from the conclusions of Steenbergen‐Hu et al. [72] on interventions for underachieving gifted students. This discrepancy likely reflects the former’s curriculum‐aligned instructional approaches, which consistently yield stronger achievement gains, in contrast to the predominantly motivational strategies (e.g., self‐regulation, goal‐setting) examined by Steenbergen‐Hu and colleagues, whose impact on performance may be less pronounced.

The second question pertained to the effect of enrichment programs on gifted students’ affective personal skills—specifically self‐concept, self‐perception, and motivation. We calculated a moderate effect size (*g* = 0.51), indicating that such programs positively influence these socio‐emotional attributes. This finding aligns with previous meta‐analytic results (*g* = 0.55) [8] and resonates with the work of Litster and Roberts [73], who reported a comparable moderate effect (*g* = 0.45) for self‐concept differences between gifted and typically developing peers. Rinn et al. [74] posit that social interaction among intellectually similar peers, facilitated by enrichment activities, bolsters personal skill development. The perceived availability of supportive peer networks within enrichment contexts [75] and the cultivation of openness to experience—a hallmark trait of gifted learners [75]—likely further enhance curiosity and socio‐emotional growth. Enrichment practices that incorporate authentic, emotionally engaging content and deep exploratory tasks may thus synergistically promote both personal and affective development.

The second research question also explored the impact of enrichment on gifted students’ affective social skills, including collaboration, entrepreneurship, and leadership. The overall effect size (*g* = 0.44) represents a small yet meaningful enhancement. Drawing on Patrick et al. [76], we suggest that the integration of problem‐ and project‐based learning, with its collaborative emphasis, underlies the observed gains in teamwork abilities. Subotnik et al. [30] further propose that talent development frameworks embedding psychosocial‐emotional self‐awareness models foster leadership competencies. Entrepreneurial skills may be nurtured through innovative enrichment environments characterized by flexibility, diverse exploratory opportunities, and process‐oriented assessment [18]. The promotion of creativity—a core element of many enrichment programs [11]—simultaneously supports entrepreneurial and leadership development. Yoon et al. [65] additionally note that tailored supervision within enrichment settings can stimulate leadership growth by aligning tasks with students’ distinct interests.

The present study also investigated the moderating role of program format on outcomes across various domains. Academic-year programs yielded the largest effect sizes, followed by summer day programs with moderate effects, which contrasts with the findings reported by Kim [8]. This discrepancy may stem from the limited number of effect sizes pertaining to summer programs in Kim’s analysis. Additionally, the divergence could be attributed to the fact that academic-year enrichment studies conducted since 2014 have increasingly focused on interventions explicitly designed to enhance cognitive skills. In contrast, socio-emotional gains in the current study exhibited effect sizes of comparable magnitude to those observed in Kim’s work, underscoring the consistent and substantial benefits of enrichment programs in supporting the socio-emotional development of gifted students.

Furthermore, this study examined the moderating influence of grade level on outcomes within cognitive and affective domains. Large effect sizes were identified across all grade levels in the present analysis (*g* = 1.34, 1.10, 1.14), whereas Kim reported a medium effect size for elementary school studies and large effects for the remaining groups (*g* = 0.57, 1.37, 2.02). These patterns suggest a broad alignment in results between the two studies. The observed differences may arise from Kim’s emphasis on academic achievement metrics (e.g., reading comprehension), as opposed to the current study’s broader focus on diverse cognitive skills (e.g., critical thinking).

When comparing socio-emotional gains, Kim identified small effects for elementary and high school levels (*g* = 0.44, 0.93, 0.29), while the present study yielded small effects for primary school, medium effects for middle school, and a combination of medium and large effects for high school (e.g., *g* = 0.21, 0.54, 1.07). Although the domains under comparison differ, the outcomes demonstrate notable convergence. Such variations can plausibly be attributed to the distinct affective constructs examined; for instance, Kim categorized variables such as attitudes and self-concept as socio-emotional, whereas the current study incorporated elements like entrepreneurship and leadership. Collectively, evidence from both investigations supports the assertion that enrichment programs meaningfully contribute to the socio-emotional development of gifted students.

## Conclusion

Over the past decade, research on enrichment practices has experienced moderate growth compared to earlier periods; nevertheless, the observed effects on skill development remain broadly consistent with those documented in previous investigations. This review highlights several nuanced insights, encompassing a detailed analysis of subdomains within academic achievement and higher-order thinking, alongside the enhancement of personal and socio-affective competencies. Taken together, these findings suggest that enrichment programs effectively foster both the cognitive and non-cognitive aspects of development among gifted learners. When considered in conjunction with Kim’s study, the body of research spanning over three decades since 1994 supports the assertion that enrichment interventions substantially bolster both academic and non-academic skills in gifted students. Several limitations of the present review warrant acknowledgment. First, the exclusive reliance on English-language publications may have constrained the breadth and representativeness of the included literature. Second, the analysis did not differentiate the varying impacts of enrichment across distinct cognitive and non-cognitive subdomains.

## Supporting information

S1 File Prisma 2020 Checklist. (PDF)

S2 File Data Set. (PDF)
