# Supplementary material for: Impacts of enrichment programs on cognitive and affective skills of gifted students: A meta-analysis
Source: PLoS One. 2025 Oct 9;20(10):e0333714. doi: 10.1371/journal.pone.0333714 (PMC12510610; doi:10.1371/journal.pone.0333714)
Supplement: S2 File — (PDF) [file pone.0333714.s002.pdf]

Data set

Study		Methodology						Effect size information									
Details	Design	Outcome	Participants		Type of Program	Sample size		Pre					Post				
				Age or grades				Mean	SD	t	p	F	Mean	SD	t	p	F
1.Batterjee, 2016	Pre-post	Self-perceptiom<br>Learning	600	4-11<br>10-18<br>years	Non	Control	267	1.83	.53				1.83<br>83.10	.58<br>11.90			
					Mawhiba (separate class)	Treatment	228	1.83	.50				1.87<br>91.20	.60<br>5.90			
2.Preckel et al, 2017	Pre-post	Academic self concept<br>Intellectual self concept	177	10-12 grades<br>14-18 ages	Summer residential school			4.66<br>4.14	.47<br>.74				4.60<br>4.00	.66<br>.82			
3. Van rossen et al. 2021	Pre-post	Social relationship with peers and teachers	429	4-6 grades	Separate class	Control	429	4.22	.69				4.20	.78			
								3.81	.69				3.76	.78			
			245			Treatment	245	4.11	.76				4.21	.67			
								3.81	.66				3.91	.62			
4. Vidergor, 2017	Pre-post	Scientific thinking<br>Creative thinking-problem solving<br>Future thinking-analytical thinking	195		Extracurricul ar enrichment program	Control		7.77<br>5.67<br>2.76	4.76<br>5.14<br>3.86				9.39<br>5.91<br>4.20	5.49<br>5.32<br>4.46			
			199			Treatment		8.30<br>5.85<br>3.80	5.68<br>4.75<br>4.90				16.71<br>20.87<br>12.04	6.92<br>6.02<br>4.46			
5. Vidergor et al., 2019	Pre-post	Future thinking	169		Extracurricul ar enrichment program	Control		1.55	3.18				2.76	3.99			
			166			Treatment		1.46	2.87				9.06	4.69			

6.Golle et al., 2018	Pre-post, Quasi experimental	Crystalized intelligence<br>Epistemic curiosity<br>Creativity<br>Self concept<br>Social competence	2751	2-3-4 grades	Non	Control	423	98.99<br>3.13<br>5.24<br>3.65<br>3.62	9.72<br>.63<br>2.17<br>.99<br>.56				104.50<br>3.05<br>5.75<br>3.45<br>3.60	9.63<br>.57<br>2.42<br>.94<br>.55			
					-a statewide extracurricular enrichment program<br>-the Hector Children's Academy Program	Treatment	2328	105.64<br>3.17<br>5.82<br>4.00<br>3.70	9.66<br>.62<br>2.24<br>.84<br>.58				110.63<br>3.19<br>6.53<br>3.96<br>3.66	9.78<br>.56<br>2.56<br>.89<br>.54			
7.Foley Nicpon et al., 2017	Pre post	Companionship<br>Conflict<br>Help<br>Security<br>Closeness	37	4-5 grades	Non	Control	9	3.44<br>2.62<br>4.00<br>3.54<br>4.11	.83<br>.95<br>.77<br>.88<br>.76				3.61<br>2.64<br>3.98<br>4.44<br>4.29	.93<br>.93<br>.65<br>.31<br>.79			
					Summer school	Treatment	28	3.28<br>2.54<br>3.84<br>3.74<br>4.25	.83<br>1.20<br>.82<br>.84<br>.74				3.51<br>2.68<br>4.04<br>4.17<br>4.29	.72<br>1.04<br>.84<br>.55<br>.68			
8. Mun and hertzog, 2018	Pre-post	Math attitude<br>Math identity	40	4-8 grades	Saturday Enrichment Program	treatment	40	3.21<br>2.72	.74<br>1.02				2.95<br>2.74	.75<br>.93			
9. Yoon et al., 2020	Pre-post	Leadership self-awareness<br>Self-confidence<br>Enhancing learning	14	9-12 grades	Youth Science and Technology	treatment	14	3.57<br>3.88<br>4.47	.45<br>.63<br>.50				4.55<br>4.77<br>4.95	.49<br>.41<br>.15			

					Leadership<br>Camp												
10.<br>Elhoweris<br>et al., 2022	Pre-post,<br>Quasi<br>experimenta<br>l	Critical reading	40	4th grade<br>9 years	Reading<br>enrichment<br>program	Control<br>Treatment	20<br>20	32.30<br>32.70	5.95<br>3.23				32.20<br>40.30	6.07<br>2.62			
11.<br>Gubbels et<br>al., 2022	Pre-post<br>control<br>group<br>experimenta<br>l	Analytical ability<br>Creative ability	46	10 years	Computer<br>based<br>enrichment<br>programme	Control<br>Treatment	20<br>26	63.0<br>67.6<br>69.62<br>67.62	14.11<br>8.02<br>6.97<br>8.92				75.15<br>66.26<br>81.80<br>64.90	18.95<br>11.01<br>8.70<br>9.52			
12.<br>Alkhuzaim<br>Al-Qutaim,<br>2022	Pre-post	İnnovative thinking<br>Curiosity<br>Perseverance<br>Imagination<br>Collaboration	42	Seconda<br>ry<br>school	Inquiry<br>based<br>enrichment<br>program	Treatment	42	3.67<br>3.81<br>3.70<br>3.50<br>3.67	.34<br>.51<br>.42<br>.57<br>.51				4.35<br>4.38<br>4.33<br>4.30<br>4.37	.29<br>.47<br>.40<br>.51<br>.44			
13. Darga<br>and<br>Ataman,<br>2021	Pre-post	Achievement	31	1st grade<br>6 years	Class wide<br>enrichment<br>program	treatment	31	17.87	6.28				36.71	5.75			
14.Ayoub<br>et al., 2022	Pre-post	Problem finding total<br>-belief identification<br>-flexible thinking<br>-dogmatic thinking<br>Openmindedness	60	8-9<br>grades	Enrichment<br>summer<br>program	treatment		129.6<br>29.20<br>63.60<br>36.80<br>4.72	12.6<br>3.82<br>6.55<br>5.97<br>4.31				133.5<br>30.58<br>65.25<br>37.70<br>11.27	10.3<br>3.84<br>5.82<br>4.99<br>9.14			

15. Muammar, 2023	Pre-post	Achievement<br>Creativity<br>Entrepreneurship<br>Leadership<br>Problem solving	430<br>125<br>114	15-17 ages	Gifted summer program	430<br>125<br>114		39.37<br>4.40<br>4.49<br>4.81<br>5.04	10.25<br>.50<br>.58<br>.52<br>.60				59.29<br>4.54<br>4.64<br>4.99<br>5.10	15.11<br>.57<br>.73<br>.59<br>.70			
16. Garcia Perales et al. 2019	Pre-post	Total	45	2-6 gardes	Extracurricular enrichment program	Treatment group	9	4.00	1.73				2.44	1.13			
						Control group with gifted	27	3.15	1.06				3.67	.87			
						Control group average student	9	4.00	1.32				5.00	1.00			
